# Supplementary material for: Convergent evolutionary patterns of heterostyly across angiosperms support the pollination-precision hypothesis
Source: Nat Commun. 2024 Feb 9;15:1237. doi: 10.1038/s41467-024-45118-0 (PMC10858259; doi:10.1038/s41467-024-45118-0)

Convergent evolutionary patterns of heterostyly across angiosperms support the pollination-precision hypothesis

Supplementary Table 1. Likelihood of the alternative models of evolution fitted to depict the macroevolutionary patterns of appearance of style-length polymorphism. AIC values of the Hidden Markov models fitted to the GBOTB tree (‘*equal rates*’ model with a single and two transition rate categories and ‘*all rates different*’ models with a single and two transition rate categories).

Model	AIC
"equal/symmetric rates" with a single transition rate category	1676.9
"equal/symmetric rates with two transition rate categories	1160.8
"all rates differ" with a single transition rate category	1245.2
"all rates differ” with two transition rate categories	1130.6

Supplementary Table 2. Likelihood of the alternative models of evolution fitted to depict the macroevolutionary correlation of style-length polymorphism with each of the six floral traits analysed. Floral traits analysed in Pagel's models with codification of alternative states, sample size (n), best fitting model of dependent evolution (x: “floral trait depends on style-length polymorphism”; y: “style-length polymorphism depends on floral trait”; xy: “style-length polymorphism depends on floral trait and vice versa”) and AIC values of the alternative models of dependent and independent evolution with style-length polymorphism. We refer in all cases to the ‘*all rates differ*’ model, which fitted the best for both the dependent and independent modes of evolution.

**Floral trait**	**state 0**	**state 1**	**n**	**Dependent model**	**AIC dependent model**	**AIC independent model**
Fusion of perianth	free (<5%)	fused (>5%)	705	x	1023.8	1052.5
Symmetry of perianth	actinomorphic	zygomorphic	652	y	769.3	775.5
Number of fertile stamens	1 to 10	more than 10	616	y	740.5	749.0
Fusion of filaments	free (<5%)	fused (>5%)	505	y	666.9	665.4
Fusion of filaments to perianth	free (<5%)	fused (>5%)	396	xy	594.2	614.6
Number of structural carpels	1 to 5	more than 5	786	x	267.2	268.1

Supplementary Table 3. Likelihood of the alternative models of evolution fitted to depict the macroevolutionary correlation of style-length polymorphism with each of the six pollination systems analysed. Pollination systems analysed in Pagel's models with codification of alternative states, sample size (n), best fitting model of dependent evolution (x: "pollination system depends on style-length polymorphism"; y: "style-length polymorphism depends on pollination system"; xy: "style-length polymorphism depends on pollination system and vice versa") and AIC values of the alternative models of dependent and independent evolution with style-length polymorphism. We refer in all cases to the '*all rates differ*' model, which fitted the best for both the dependent and independent modes of evolution. In the analyses of *long-tongued insects* pollination system, we report both x and y models of dependent evolution as they received similar support.

**Pollination system**	**state 0**	**state 1**	**n**	**Dependent model**	**AIC dependent model**	**AIC independent model**
Biotic	abiotic	biotic	1495	y	572.3	572.8
Insect (vs. vertebrate)	vertebrate	insect	1433	x	1019.4	1015.1
Insect (vs. birds)	bird	insect	1413	x	901.6	901.5
Specialised	generalist	specialist	1271	y	1911.0	1909.1
Long-tongued animals	short-tongued	long-tongued	621	y	814.1	801.9
Long-tongued insects	short-tongued	long-tongued	512	x	680.3	731.0
Long-tongued insects	short-tongued	long-tongued	512	y	680.9	731.0

Supplementary Figure 1. Correlated evolution of style-length polymorphism and biotic and insect pollination systems. Graphic representation of Pagel's models of dependent evolution of style-length polymorphism (represented through two morphs state) on 'biotic' (red) vs. 'abiotic' (blue) pollination systems (A) and on 'insect' (red) vs. 'bird' (blue) pollination systems (B). The state with the highest association with style-length polymorphism is on the top left. These models presented similar support than the corresponding model of independent evolution.

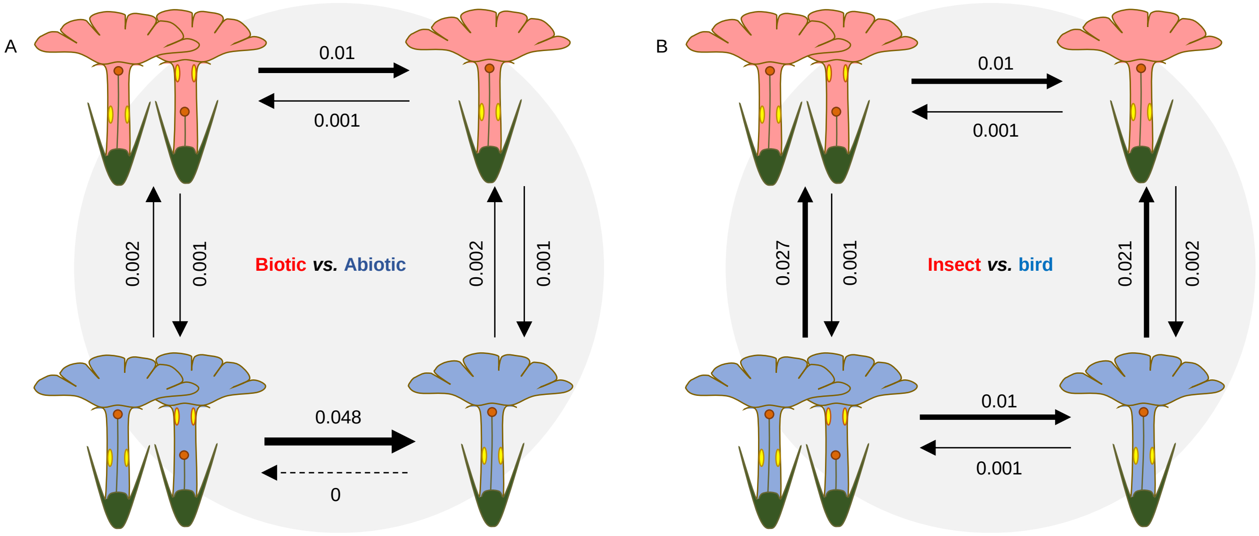

Supplement: Supplementary file 1 — Supplementary Information [file 41467_2024_45118_MOESM1_ESM.pdf]
