# Supplementary material for: Convergent evolutionary patterns of heterostyly across angiosperms support the pollination-precision hypothesis
Source: Nat Commun. 2024 Feb 9;15:1237. doi: 10.1038/s41467-024-45118-0 (PMC10858259; doi:10.1038/s41467-024-45118-0)
Supplement: Supplementary file 3 — Description of Additional Supplementary Files [file 41467_2024_45118_MOESM3_ESM.pdf]

Description of Additional Supplementary Files

File Name: Supplementary Data 1
Description: Style-length polymorphic genera database including references, type of report, links to dioecy, and doubtful genera.

File Name: Supplementary Data 2
Description: Original data records and compiled matrices of floral traits extracted from the PROTEUS database.

File Name: Supplementary Data 3
Description: Database on the pollination system of style-length polymorphic species, references for the pollination system across all angiosperm species, and database on the pollination system of the species included in the GBOTB tree.
