# Supplementary material for: Convergent evolutionary patterns of heterostyly across angiosperms support the pollination-precision hypothesis
Source: Nat Commun. 2024 Feb 9;15:1237. doi: 10.1038/s41467-024-45118-0 (PMC10858259; doi:10.1038/s41467-024-45118-0)
Supplement: Supplementary file 7 — Reporting Summary [file 41467_2024_45118_MOESM7_ESM.pdf]

nature portfolio

Corresponding author(s): Simón-Porcar, Violeta I., Arroyo, Juan.

Last updated by author(s): 2023/11/30

# Reporting Summary

Nature Portfolio wishes to improve the reproducibility of the work that we publish. This form provides structure for consistency and transparency in reporting. For further information on Nature Portfolio policies, see our Editorial Policies and the Editorial Policy Checklist.

## Statistics

For all statistical analyses, confirm that the following items are present in the figure legend, table legend, main text, or Methods section.

| n/a | Confirmed | |
|---|---|---|
| ☐ | ☒ | The exact sample size (*n*) for each experimental group/condition, given as a discrete number and unit of measurement |
| ☒ | ☐ | A statement on whether measurements were taken from distinct samples or whether the same sample was measured repeatedly |
| ☒ | ☐ | The statistical test(s) used AND whether they are one- or two-sided<br>*Only common tests should be described solely by name; describe more complex techniques in the Methods section.* |
| ☐ | ☒ | A description of all covariates tested |
| ☐ | ☒ | A description of any assumptions or corrections, such as tests of normality and adjustment for multiple comparisons |
| ☐ | ☒ | A full description of the statistical parameters including central tendency (e.g. means) or other basic estimates (e.g. regression coefficient) AND variation (e.g. standard deviation) or associated estimates of uncertainty (e.g. confidence intervals) |
| ☒ | ☐ | For null hypothesis testing, the test statistic (e.g. *F*, *t*, *r*) with confidence intervals, effect sizes, degrees of freedom and *P* value noted<br>*Give P values as exact values whenever suitable.* |
| ☒ | ☐ | For Bayesian analysis, information on the choice of priors and Markov chain Monte Carlo settings |
| ☒ | ☐ | For hierarchical and complex designs, identification of the appropriate level for tests and full reporting of outcomes |
| ☒ | ☐ | Estimates of effect sizes (e.g. Cohen's *d*, Pearson's *r*), indicating how they were calculated |

*Our web collection on statistics for biologists contains articles on many of the points above.*

## Software and code

Policy information about availability of computer code

| | |
|---|---|
| Data collection | All new data was collected from the scientific literature using the Google Scholar specialized search engine. We used the Plants of the World database (powo.science.kew.org) for taxonomic correction of genera names. We used the PROTEUS database of floral morphological traits, assembled within the eFLOWER initiative (Sauquet et al., 2017). We used the species-level phylogenetic tree for seed plants published by Smith and Brown (2018) ('GBOTB tree'). |
| Data analysis | We used the  function drop.tip of the R package ape v5.6. (Paradis et al., 2004); the functions corHMM and makeSimmap of the R package corHMM v2.8. (Beaulieu et al., 2017);  the function fitPagel of the R package phytools v1.5.1. (Revell, 2012); the function ltt.plot of the R package ape v5.6. (Paradis et al., 2004); the function ggplot of the R package ggplot2 v3.4.1. (Wickham, 2016); and the function locmodes of the R package multimode v1.5. (Ameijeiras-Alonso et al., 2018). |

For manuscripts utilizing custom algorithms or software that are central to the research but not yet described in published literature, software must be made available to editors and reviewers. We strongly encourage code deposition in a community repository (e.g. GitHub). See the Nature Portfolio guidelines for submitting code & software for further information.

## Data

Policy information about availability of data

All manuscripts must include a data availability statement. This statement should provide the following information, where applicable:

- Accession codes, unique identifiers, or web links for publicly available datasets
- A description of any restrictions on data availability
- For clinical datasets or third party data, please ensure that the statement adheres to our policy

All data supporting the findings of this study are available within the paper and its Supplementary Information.

## Research involving human participants, their data, or biological material

Policy information about studies with human participants or human data. See also policy information about sex, gender (identity/presentation), and sexual orientation and race, ethnicity and racism.

| | |
|---|---|
| Reporting on sex and gender | This study does not involve human research participants |
| Reporting on race, ethnicity, or other socially relevant groupings | This study does not involve human research participants |
| Population characteristics | This study does not involve human research participants |
| Recruitment | This study does not involve human research participants |
| Ethics oversight | This study does not involve human research participants |

Note that full information on the approval of the study protocol must also be provided in the manuscript.

# Field-specific reporting

Please select the one below that is the best fit for your research. If you are not sure, read the appropriate sections before making your selection.

☐ Life sciences ☐ Behavioural & social sciences ☒ Ecological, evolutionary & environmental sciences

For a reference copy of the document with all sections, see nature.com/documents/nr-reporting-summary-flat.pdf

# Ecological, evolutionary & environmental sciences study design

All studies must disclose on these points even when the disclosure is negative.

| | |
|---|---|
| Study description | We carried out a systematic literature search on Google Scholar to update the number of style-length polymorphic genera. We compiled a data set of floral traits related to pollination precision across all angiosperm families, using the PROTEUS database of floral morphological traits, assembled within the eFLOWER initiative. We carried out a systematic literature search on Google Scholar to compile a database on the pollination system of polymorphic and non-polimorphic angiosperm species. We used a megaphylogeny of Angiosperm genera to model the evolution of style-length polymorphism and to analyze its evolutionary associations with the floral traits related to pollination precision. |
| Research sample | The research sample is the whole Angiosperm clade, to which style-length polymorphism is restricted by definition. To obtain a complete list of style-length polymorphic genera, we used the review of Naiki (2012) as a backbone, which included 199 heterostylous genera. To obtain a global angiosperm phylogeny at the genus level, we used the species-level phylogenetic tree for seed plants published by Smith and Brown (2018) ('GBOTB tree'), which includes nearly 10,000 genera. To obtain a dataset of floral traits related to pollination precision, we used the last published angiosperm-wide eFLOWER dataset (Schönenberger et al., 2020) as a backbone. This dataset included data for 29 primary floral characters in 792 species from 776 genera, 372 families (86%) and 63 out of 64 angiosperm orders recognized in APG IV (2016). We compiled data on the pollination system of 5038 angiosperm species, from which 1495 were included in the GBOTB tree. |
| Sampling strategy | We followed a maximum sampling strategy. We virtually listed all existing records of style-length polymorphism, and used the biggest existing assembled phylogeny of Angiosperm genera and database of floral traits. We assembled, to our knowledge, the lasrgest dataset on pollination system of angiosperm species. We consider genera as the lower taxonomic level possible for our analyses, as it is currently impossible to get an approximate estimate of the distribution of style-length polymorphism at the species level. |
| Data collection | VISP updated the list of style-length polymorphic genera by performing a systematic search on Google Scholar without publishing year limitations. VISP expanded the PROTEUS floral traits database with data for 10 floral traits related to pollination precision in 231 species from different genera, following the standard scoring methodology of the eFLOWER initiative (Sauquet et al., 2017; Schönenberger et al., 2020). VISP compiled the pollination system database by performing a systematic search on Google Scholar without publishing year limitations. |

| | |
|---|---|
| Timing and spatial scale | The systematic search of style-length polymorphic genera was performed in November 2020 and updated in January 2022. The floral traits scoring was performed in August 2021. The systematic search of pollination systems was performed in October 2023. |
| Data exclusions | Four scored floral traits were excluded from the comparative analyses because of their low number of available records and/or their uneven distribution in style-length polymorphic and monomorphic genera. |
| Reproducibility | The reproducibility of this study is complete following the methods described and using the data provided as supplementary material. The full pollination system database is available upon request for reproducibility purposes. |
| Randomization | This is not relevant for this study as it does not include experimental plots. |
| Blinding | Blinding is not relevant for this study, as we followed a systematic strategy to score the maximum number of records possible in every case. |

Did the study involve field work? ☐ Yes ☒ No

# Reporting for specific materials, systems and methods

We require information from authors about some types of materials, experimental systems and methods used in many studies. Here, indicate whether each material, system or method listed is relevant to your study. If you are not sure if a list item applies to your research, read the appropriate section before selecting a response.

## Materials & experimental systems

| n/a | Involved in the study |
|---|---|
| ☒ | ☐ Antibodies |
| ☒ | ☐ Eukaryotic cell lines |
| ☒ | ☐ Palaeontology and archaeology |
| ☒ | ☐ Animals and other organisms |
| ☒ | ☐ Clinical data |
| ☒ | ☐ Dual use research of concern |
| ☒ | ☐ Plants |

## Methods

| n/a | Involved in the study |
|---|---|
| ☒ | ☐ ChIP-seq |
| ☒ | ☐ Flow cytometry |
| ☒ | ☐ MRI-based neuroimaging |

## Plants

| | |
|---|---|
| Seed stocks | This study does not involve plant material |
| Novel plant genotypes | This study does not involve plant material |
| Authentication | This study does not involve plant material |
